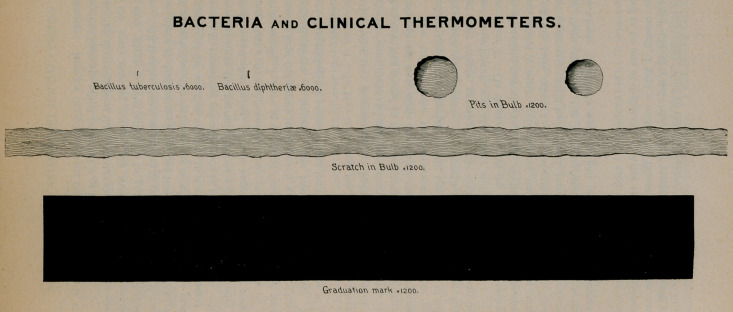# The Clinical Thermometer as a Germ Carrier1Read at the thirty-second annual meeting of the Medical Association of Central New York, at Syracuse, October 17, 1899.

**Published:** 1900-02

**Authors:** W. L. Conklin

**Affiliations:** Rochester, N. Y.


### BUFFALO MEDICAL JOURNAL.

VOL. XXXIX.—LV. FEBRUARY, 1900. No. 7.

### Original Communications.

### THE CLINICAL THERMOMETER AS A GERM CARRIER.[1]

BY W. L. CONKLIN, M. D., Rochester, N. Y.

IT IS safe to say that no other article of the physician's outfit is used so constantly, and in such a variety of diseases as the clinical thermometer. In exceptional cases, it is true, the information which it furnishes is of little value, but in all contagious diseases, and most others, the service which it renders as an aid to diagnosis and treatment is so evident that its use has come to be a routine practice with us all.

This frequent and promiscuous use of the thermometer furnishes food for thought when we reflect that in a large proportion of cases the temperature is taken in the mouth, which is the abiding place of innumerable germs, and that many of these germs are capable of producing morbid conditions which endanger life itself.

We rightly consider absolutely sterile instruments and dressings and surgically clean hands, as requisites to successful operative work; and to open an abscess for one patient and a vein for the next with a scalpel which had been cleaned, if cleaned at all, by wiping it on a towel, would be inexcusable disregard of a fundamental rule of surgical practice. But are we equally careful to see to it that our thermometers are bacteriologically clean? And if not, *why not?* Assuming that the thermometer, as well as the scalpel, may be a germ carrier, does not the mouth furnish as favorable a medium for the development and growth of bacteria as the open wound?

---

1. Read at the thirty-second annual meeting of the Medical Association of Central New York, at Syracuse, October 17, 1899.

There is evidently an important difference between the cleaning which destroys germs and renders an object surgically or bacteriologically clean, and the cleaning which is effective only so far as the removal of macroscopic objects is concerned. Is it not the latter process, rather than the former, to which our thermometers are subjected? It has been asserted that the ordinary methods of cleaning a thermometer, such, *e.g.*, as holding it under the water faucet, or wiping it with a damp cloth are sufficient to rid it of bacteria. That such is not the case, however, is conclusively shown, I think, by the chart which I present, together with the reports of bacteriological examinations made for me by Prof. Charles Wright Dodge, of the University of Rochester. The chart is an enlargement of a camera drawing, made by Professor Dodge, and represents, with mathematical exactness, the size of a tubercle bacillus and a Klebs-Löffler bacillus, as compared with the length and width of the shorter degree marks on a thermometer. It also gives us an accurate picture of a scratch and pits found with the microscope in the glass of the thermometer bulb. These are not visible to the naked eye, but the microscope reveals multitudes of them. A glance at the chart will show that they are large enough to harbor microörganisms galore.

You will observe that the degree mark and these scratches and pits are magnified only 1,200 diameters, while the bacilli are magnified 6,000 diameters. If the degree mark were magnified in the same proportion as the bacilli, it would be over thirty feet long and nearly four feet wide. A simple computation, based upon the accurate measurements of the camera drawing, shows that a degree mark is wide enough to accommodate 100 tubercle bacilli, marching in single file, so to speak. Also that an area with the length and breadth of one of these marks would furnish room for the lodgement of 280,000 tubercle bacilli. When we consider that degree marks have depth, which cannot, however, be readily measured, we see that each of them is capable of harboring a much larger number than this. These figures are of practical value, so far as they help us to realise the extreme minuteness of microörganisms as compared with the indentations, visible and invisible, on the surface of our thermometers. It seems evident that ordinary washing and wiping can not be relied on to dislodge bacilli from a surface covered with such indentations.

The chart is offered in evidence, as the lawyers say, by way of proving the possibility of making a germ carrier out of the clinical

### BACTERIA AND CLINICAL THERMOMETERS.

Bacillus tuberculosis x6000. Bacillus diphtheriæ x6000.

Pits in Bulb x1200.

Scratch in Bulb x1200.

Graduation mark x1200.

thermometer. Now for the actual proof that a germ carrier it is, if the ordinary methods are relied on to rid it of bacteria.

Believing that a clinical thermometer should at all times be sterile, I have for some months carried my thermometer in an ordinary rubber case, which is filled with a 1 to 500 or 1 to 250 bichloride solution. All that has been necessary to prevent leakage of the solution is a piece of leather packing, but there is gradual shrinkage in amount as each time the thermometer is withdrawn from the case a small portion of the solution adheres to it and I find it necessary on this account to renew the solution once in three or four days. It is my custom to rinse the thermometer in a glass of water or under the faucet before and after using. While experimenting for the purpose of proving that a thermometer cleaned in the ordinary way is not necessarily sterile, I have at the same time sought for proof that a thermometer kept constantly immersed in a strong bichloride solution, as described above, is entirely free from microörganisms. The results of bacteriological examinations for these two purposes will be given together.

*Experiment A.*—In May last I took the temperature of a tubercular patient with two thermometers. Both were then washed by pouring water over them and one was returned to an ordinary case, while the other was placed in the case containing 1 to 500 bichloride. Petri dish cultures were made from both thermometers by Professor Dodge. The culture from the thermometer which had not been sterilised developed no tubercle bacilli, but several colonies of other microörganisms were found. No growth whatever could be found from the other culture, proving that the thermometer which had been immersed in bichloride was absolutely sterile.

*Experiment B.*—On September 4th the temperature was taken in the case of a child convalescing from diphtheria. The thermometer was then rinsed in water, rubbed over the blood serum of a culture tube and the tube taken to the laboratory of the health board. While no diphtheria bacilli were found, throat germs were abundant, some of them showing the characteristics of the staphylococcus pyogens albus. Evidently washing had not rid the thermometer of germs.

*Experiment C.*—On October 3d, I took the temperature of a case suspected to be one of diphtheria. After removing the thermometer from the mouth, I held it under the faucet, washing it in the usual way, I then rubbed it over the blood serum as in experiment B and returned the culture tube to the laboratory.

Following is the report of Professor Dodge: "Microscopic examination showed presence of many involution forms of diphtheria bacilli, together with a number of other forms of bacteria not identified, but frequently found in throat cultures."

Through the kindness of Dr. Kiefer I have placed a slide from this thermometer culture under the microscope for the inspection of any who may be interested. Those organisms showing irregularity of outline and staining are good specimens of Klebs-Löffler bacilli.

*Experiment D.*—On October 5th, through the courtesy of the house-physician of St. Mary's Hospital, I took the temperature of a convalescing case of diphtheria, using two thermometers. After taking the temperature, both thermometers were held under the faucet and thoroughly washed. One of them was then rubbed over the blood serum of a culture tube and the tube marked "A." The other was placed in its case, which contained a 1 to 250 bichloride solution. One half hour later this thermometer was removed from its case, held under the faucet again to rinse off any bichloride solution which might adhere to it, and then rubbed over the blood serum of a culture tube, which was marked "B."

On the next day Professor Dodge reported as follows: "Culture tube marked "A" showed no diphtheria bacilli, but an abundant growth of staphylococcus pyogens albus and numerous diplococci. Culture tube, marked "B," furnished absolutely no growth of any kind.

To summarise: Bacteriological examination of six thermometers. Four had been washed but not sterilised. Microörganisms, of one or another variety, were found on each of the four. Two had been washed and then placed in a case containing bichloride solution. No microörganisms were found on either.

If these experiments furnish conclusive proof, and I think they do, that the thermometer may be a germ carrier, if cleaned in the ordinary way; and if they show, further, that by means of a very simple and inexpensive device it may be rendered sterile after each use, may I not, as the conclusion of the whole matter, urge the importance of using, always and only, a thermometer which is bacteriologically clean?

232 South Avenue.

### DISCUSSION.

Dr. Sears.—I learned yesterday a circumstance which would lead us to believe that it would not be a bad idea for some of our school teachers to take lessons in bacteriology. I discovered that a teacher in the primary department is allowing the children to blow soap bubbles from the same pipe indiscriminately. If there is anything worse than that to disseminate disease, I don't know anything in my experience that equals it. And, in fact, not only that, but there were a number of cases of scarlet fever and diphtheria in the school district. I immediately wrote the principal in that district, asking if there was any way of instructing the teachers in bacteriology so that this thing would not occur again. That is one of the most flagrant lacks of knowledge that I think ever came to my notice.

Dr. CLIFFORD MERCER.—I think quite recently—it may still be true—pencils have been passed in the public schools in Syracuse, to be used by the pupils, and they commonly chew the ends. They are gathered up. The next time they are repassed and they go to another child. And this was done repeatedly, day after day. I believe that goes one step further.

Dr. VAN DE WARKER.—That isn't anything. I had occasion to visit officially a truant school. Went into the bath rooms. Tumbler on the shelf. Toothbrush sticking out of it. And I said to the custodian, "whose toothbrush is that?" "He said, it belongs to the boys; we inculcate habits of cleanliness." There were eighteen boys in the truant school at that time.

Dr. CLIFFORD MERCER.—But to the original thought: it has been a source of a great deal of annoyance to my conscience, in every way, to occasionally be present while men have taken temperatures in contagious cases among children, men older than myself sometimes, who make no attempt whatever at washing the thermometer. They use it upon the subject and put it in their case with no cleanliness whatever. What can you say? That is a thousand miles away from this idea of keeping it in a vat; but men do that. They do it every day. I have seen them do it. I couldn't say anything, but if they are here, they may know whom I mean.

Dr. HOWE, Ontario.—It has been my custom for several years, as I presume it has also of many others, to keep quite a number of thermometers. I never use a thermometer continually in my practice. I think that every practitioner ought to have quite a collection of thermometers. I think that the remarks of Dr. Conklin are practical, sensible and timely, and that it would seem to me that we ought to assist in encouraging the profession to follow out just the lines suggested by him. Of course, if we could have this little reservoir, in which our thermometer could be continually carried, which I have not, that would simplify matters and we would get along with far fewer thermometers. But, in my case, I have always made it a practice, as I say, of keeping on hand quite a number of clinical thermometers and I think that too great care cannot be given to them, and I think it is the duty of all of us to listen attentively and to follow the suggestions thrown out by Dr. Conklin, which are so timely and wise.

Dr. CONKLIN.—I want to say that one point is the simplicity of the device which I have. All you have to do is to pour some bichloride into your ordinary hard rubber thermometer case and put a little packing around the cap and you have it, and such a thermometer has been proved, by two careful bacteriological examinations, to be absolutely sterile. I think it is so easy to have a sterile thermometer at all times that we ought never to use one which may be a carrier of disease.

## Figures and Tables

**Figure f1:**